# Effect of Exogenous Cues on Covert Spatial Orienting in Deaf and Normal Hearing Individuals

**DOI:** 10.1371/journal.pone.0141324

**Published:** 2015-10-30

**Authors:** Seema Gorur Prasad, Gouri Shanker Patil, Ramesh Kumar Mishra

**Affiliations:** 1 Center for Neural and Cognitive Sciences, University of Hyderabad, Hyderabad, India; 2 AliYavar Jung National Institute for the Hearing Handicapped, Secunderabad, India; Tsinghua University, CHINA

## Abstract

Deaf individuals have been known to process visual stimuli better at the periphery compared to the normal hearing population. However, very few studies have examined attention orienting in the oculomotor domain in the deaf, particularly when targets appear at variable eccentricity. In this study, we examined if the visual perceptual processing advantage reported in the deaf people also modulates spatial attentional orienting with eye movement responses. We used a spatial cueing task with cued and uncued targets that appeared at two different eccentricities and explored attentional facilitation and inhibition. We elicited both a saccadic and a manual response. The deaf showed a higher cueing effect for the ocular responses than the normal hearing participants. However, there was no group difference for the manual responses. There was also higher facilitation at the periphery for both saccadic and manual responses, irrespective of groups. These results suggest that, owing to their superior visual processing ability, the deaf may orient attention faster to targets. We discuss the results in terms of previous studies on cueing and attentional orienting in deaf.

## Introduction

Auditory deprivation in the deaf leads to compensatory changes in the attentional and perceptual systems [1], [2]. Deaf individuals can detect visual targets faster compared to the normal hearing participants, especially at the visual periphery [3], [4]. This perceptual advantage in the deaf individuals has been linked to their superior visual span that extends into the periphery [5]. Apart from a noticeable advantage in visual processing, the deaf have also been shown to have a better attentional orienting mechanism (see [6] for a review, [7],[8]). However, there are still inconsistencies in findings on spatial orienting of attention in the deaf [9]. Additionally, very few studies have examined eye movement responses in spatial cueing tasks in the deaf. Therefore, it remains to be examined if deaf and normal hearing individuals differ with regard to facilitation and inhibition in a spatial cueing task when ocular responses are measured, particularly when targets appear in the periphery. In this study, we measured saccadic and manual responses in a spatial cueing task in deaf and normal hearing when targets appeared at perifovea and periphery.

Posner's cost and benefit paradigm has been widely used to study the mechanisms related to attentional orienting in different populations through endogenous and exogenous cueing [10]. Participants are faster in detecting a target at a cued location if it appears immediately (<100ms) after cue offset.This facilitation is an outcome of faster orienting of attention towards the cued location. However, responses become slower to targets that appear at cued locations after a delay (SOA > 300ms), which has been termed as inhibition of return (IOR) [10],(see [11] for a review). Therefore, in a spatial cueing paradigm behavioural facilitation and inhibition depends on the temporal delay between cue offset and target onset. Since the deaf show superior alertness to visual stimuli, it is reasonable to assume that the differences between the deaf and normal hearing should be more robust for exogenous cueing but not for endogenous cueing. This is because exogenous cues capture attention involuntarily whereas moving the spotlight of attention in endogenous cues is voluntary.

Does the location of targets influence spatial orienting in cueing tasks? We measured ocular responses to cued and uncued targets when they appeared at perifovea (7 degree) and periphery (17 degree). Previous studies have shown that target eccentricity can influence the distribution of reaction times on a spatial cueing task [12]. Inhibitory effects are stronger when targets appear in the periphery compared to the perifovea [13]. Therefore, attentional effects resulting in facilitation and inhibition can vary from perifovea to the periphery in a cueing task. Bao et al. [14] studied the effect of eccentricity on IOR in hearing individuals with the cueing task and found that the magnitude of IOR was higher at the periphery compared to the perifovea. If the deaf can attend to visual stimuli at the periphery better, then it is likely that the deaf also will show superior attentional orienting mechanisms at the periphery compared to the hearing when targets appear at two eccentricities. In a manual cueing task, Proksch and Bavelier [15] found higher interference with irrelevant peripheral distracters in the deaf compared to the hearing individuals. That is, the deaf were more sensitive to distractors that appeared at the periphery than at central locations. This could be explained considering that the deaf have a superior visual perceptual span and show higher sensitivity to stimuli that appear at the periphery.

The studies that have examined attentional orienting in the deaf using the spatial cueing paradigm have found a divergent pattern of results [3], [16], [17]. While some have observed an overall RT advantage for the deaf in such tasks [4], others have observed the faster decay of inhibition where longer SOAs have been used[3]. Yet, some other studies have not observed any group difference in inhibition between the deaf and the hearing individuals [18]. It is important to note that while a faster orientating of attention can explain observed facilitation at the cued locations at shorter SOAs, inhibition at longer SOAs is contingent upon several other factors. There have been conflicting reports about the appearance of inhibition in the deaf in cueing tasks [3], [18]. In a spatial cueing task with exogenous cues, inhibition at the cued location for valid trials arises when attention is first disengaged and does not return to that location. It has been also suggested that inhibition of return indicates our need to forage visually the environment for new information rather than returning to the visited locations. Klein (2000) suggested that the time course of IOR depends on the efficiency of withdrawing attention from the cued location. Action video game players have been shown to have earlier inhibition of return than non-video game players [19]. On the other hand in patients with Schizophrenia, the appearance of IOR is delayed compared to normal healthy participants [20]. This is possibly linked to slower disengagement of attention from the cued location.

If one assumes that deaf disengage attention from the cued location rapidly, then one should expect an earlier appearance of IOR in the deaf compared to normal hearing participants. In an earlier study with spatial cueing in the deaf, Colmenero et al. [3] observed early appearance of IOR in the deaf suggesting rapid disengagement of attention from the cued location. Colmenero et al. [3] presented peripheral cues at 14-degree eccentricity and measured responses to targets in a detection task in deaf and hearing individuals. The deaf were not particularly faster in responding to targets that appeared at cued locations but showed a reduced cost i.e. delay in responding when the target appeared at an uncued location (Experiment 1). They also observed that the deaf participants displayed a faster rate of decay in inhibition of return (Experiment 2). However, Xingjuan et al. [17] had obtained contrasting results in a similar paradigm. Although they found that IOR developed faster in deaf at short SOA (350 ms), there was no group difference as far as decay of IOR was concerned. In yet another study, Chen et al. [18] presented central targets and peripheral distractors in a detection task and observed comparable IOR at long SOA (900 ms) in the deaf and the hearing. Therefore, while differences in facilitation in deaf and hearing individuals have been observed in spatial cueing tasks with shorter SOAs, no group difference in inhibition has been consistently observed at longer SOAs. The differences between the studies may have arisen because of different methods and paradigms. It is quite possible that disengagement of attention and its speed is task contingent. Therefore, it is not clear if an excellent visual processing advantage seen in the deaf should influence both facilitation at shorter SOAs and inhibition at longer SOAs similarly for targets that appear at different eccentricities.

In this study we measured both ocular and manual responses. Most of the above-reviewed studies that have compared the visual attentional abilities between deaf and hearing individuals using different variations of the cueing paradigm have measured manual responses [4], [16], [18], [3], [17]. No previous study (to the best of our knowledge) has measured saccadic responses in a spatial cueing paradigm in the deaf. Therefore, how deafness modulates attentional facilitation and inhibition when ocular responses are measured remains unexplored. Saccades have been known to share a close functional relationship with the attentional and visual systems [21]. Therefore, the visual perceptual advantages observed in the deaf should modulate saccadic responses to a greater extent than the manual responses. It has been shown that facilitation and inhibition seen in cueing tasks may manifest differently for manual and ocular responses [22], [23]. Briand et al. [22] had observed that inhibition appears earlier in the ocular responses compared to the manual. In one earlier study where eye movements were measured in an attentional task, Bottari et al. [24] used the pro- and anti-saccade task to study reflexive and voluntary eye movements in deaf and hearing individuals and manipulated target eccentricity. They found faster latencies and fewer errors in the pro-saccade trials compared to anti-saccade trials. Additionally, this "facilitation" was found to be larger in the deaf compared to the hearing. But, the authors did not find an effect of target eccentricity on oculomotor responses that they attribute to the blocking of trials with a specific eccentricity and/or to the limited eccentricity of their peripheral targets. Therefore, it is not clear, if the deaf should show superior ocular orienting in a spatial cueing task when targets appear at the periphery.

In this study, a group of congenitally deaf signers and age-matched hearing participants participated in a spatial cueing task where the targets appeared at two different eccentricities i.e. 7 and 17 degrees. We considered 7-degree eccentricity as perifovea and 17 degree as periphery (but see [14]). We asked participants to make an eye movement when a target appeared either to the left or the right of the central fixation at different spatial eccentricity and followed by a manual response. The peripheral cues were unpredictive with regard to the targets. We expected an influence of the target's eccentricity on the cueing effect differently for the deaf than the normal hearing participants. This conjecture follows from earlier reports of superior visual processing to stimuli appearing at the peripheral locations in the deaf [7], [8]. We used both shorter and longer SOAs to examine facilitation and eventual inhibition in target detection. While facilitation may be a result of speedy attentional orientating because of the abrupt onset of a peripheral cue, inhibition (slower responses to targets appearing at cued locations at longer cue-target SOA) can be because of disengagement of attention [25]. Therefore, if the superior visual perceptual advantage in the deaf also influences rapid attentional disengagement from the cued location, we should observe lesser inhibition in the deaf at the periphery compared to the hearing individuals. Also, following Bao et al., [14], one should expect a larger cueing effect at the periphery compared to perifovea, regardless of the groups.

## Method

### Participants

Fifteen right-handed deaf individuals, all male (Mean age = 24.2, SD = 6.46) and fifteen right handed hearing students, (four females, Mean age = 25, SD = 6.68 and 11 males, Mean age = 26.72, SD = 6.40) participated in the study. All the hearing-impaired participants were congenitally deaf, were born to hearing parents and had a profound sensorineural hearing loss in both the ears. We circulated a questionnaire to all the deaf participants that had questions related to their medical history and also sign language use (See Table 1). All of them used Indian Sign Language (ISL) which they reported having acquired at an average age of 5 years. All participants had normal or corrected-to-normal vision. None of the participants reported any neurological disease. The deaf participants had at least 12 years of education in a special school/college set up that educated the deaf. Thus, all of them were proficient users of Indian sign language which was their medium of instruction and also communication. The hearing individuals were students from the University of Hyderabad. The ethics committee of University of Hyderabad approved this research. Written consent was obtained from all participants through a consent form approved by the ethics committee. No minors or children participated in the study.

**Table 1:** Deaf participant details.

Subject	Duration of use of hearing devices (in years)	Age of acquisition of sign language (in years)
D1	NA	9
D2	10	5
D3	9	5
D4	5	4
D5	5	6
D6	8	4
D7	6	5
D8	5	3
D9	6	3
D10	4	4
D11	8	6
D12	4	5
D13	5	5
D14	5	4
D15	5	5

Note: All deaf participants were congenitally deaf born to hearing parents and suffered from profound sensorineural hearing loss. They received education in special schools for the deaf in which the primary medium of communication was Indian Sign Language. They were asked to rate their sign language proficiency on a 3-point scale (1 –Low proficient, 2 –moderately proficient, 3 –high proficient). All the participants reported high proficiency (a rating of 3), NA–Not applicable

### Apparatus and Procedure

**Fig 1:**
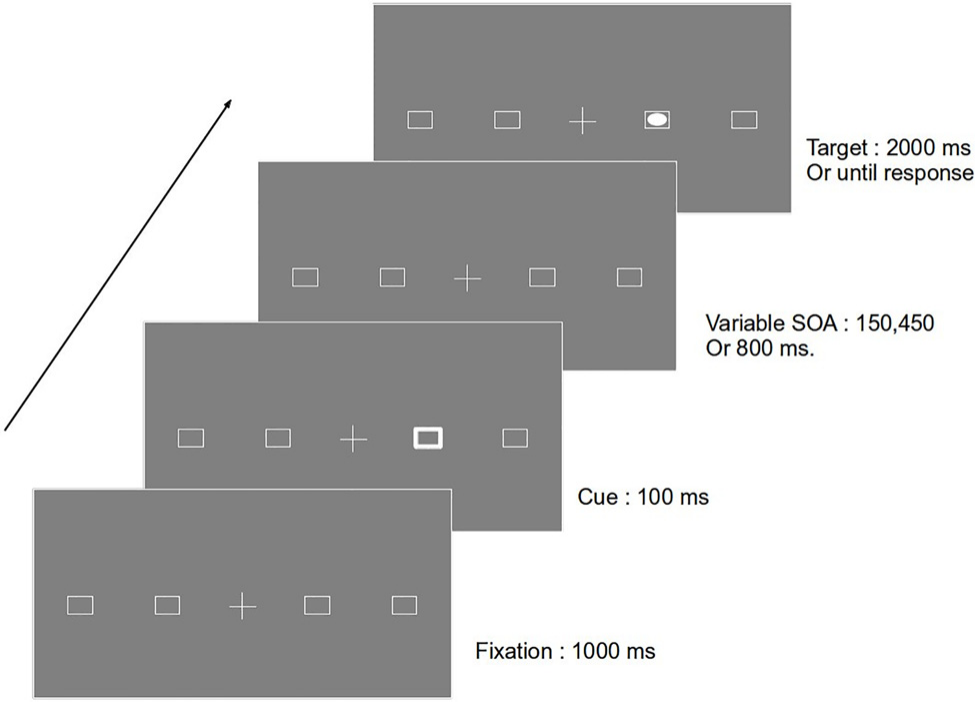
Sequence of events in a sample trial. In this example there is an exogenous cue at 7 degree eccentricity. After a variable SOA, the target appears at the same location and stays till a manual response is made (or 2000 ms, whichever is the earliest). This is considered as a valid trial.

The experiment took place in a noise free and dimly lit room. Participants sat at a distance of 60 cm from a 19 inch LCD monitor with a screen resolution of 1024 × 768 pixels and refresh rate of 60 Hz. Stimuli were prepared using the SR Research Experiment Builder software and collection of eye movement data was controlled by a computer running Eyelink 1000 software with a sampling rate of 1000 Hz. (SR Research Ltd, Ontario, Canada). Each trial began with a white fixation cross subtending an angle 1° × 1° and two white boxes on either side of the cross. The background was grey throughout the experiment. The boxes 1° × 1° were placed horizontally at an eccentricity of 7° (left, right) and 17° (left, right). After 1000 ms, the outline of one of the boxes thickened for 100 ms serving as a peripheral cue. The target appeared inside one of the boxes after a variable stimulus onset asynchrony(SOA) of 150 ms, 450 ms and 800 ms. The target was a white disc subtending 0.6° × 0.6° which remained for 2000 ms. The inter-trial interval was of 1000 ms (refer Fig 1).

The experiment had 960 trials that were administered in 8 blocks. The trials were equally divided between the two eccentricities and the three SOA's. Each block had 120 trials with 48 valid and 48 invalid trials. On valid trials, the target appeared in the cued box after a specified SOA. On invalid trials, the targets appeared on the side opposite to the cue, but at the same eccentricity as the cue. The remaining twenty-four were catch trials in which no target appeared and the participant was instructed to refrain from making any response. The participants were instructed to start every trial by looking at the fixation cross at the centre of the monitor which was gaze-contingent. The trial only began after the participants fixated for a minimum of 1000 ms at this fixation cross. The participants were then required to make a saccade towards the target and also make a manual response using the "SPACE" key as soon as they detected the target. Participants were also told that cues may not predict the appearance of the target and therefore, they should not make eye movements to the cues. There were breaks after every two blocks. Twenty practice trials were given before the start of the experiment.

### Data analysis

The eye movement data was extracted using the Dataviewer analysis software (SR Research, Ontario). The continuous eye movement record was analyzed for saccades following a velocity based algorithm that defined saccadic events as eye movements in any direction from a fixation with a velocity of 30°/s. For each trial, the first saccade that originated after the appearance of the target was considered for analysis. Saccade latency was defined as the time taken to initiate a saccade after target onset.

## Result

### Saccade Latency

We removed a total of 19.2% (16.8% in deaf and 21.6% in hearing) of the trials where saccadic latency was below 80 ms and above 1000 ms. An independent samples t test revealed that the percentage of trials excluded based on RT criteria was not significantlydifferent for the deaf compared to the hearing, *t* (28) = 1.11, *p* = 0.27. Saccades that didn’t originate from a square of side measuring 2° around the fixation cross were also excluded from analysis. The percentage of such trials was 12.37% in the deaf group and 13.03% in the normal hearing group. This difference (M = 0.66, SD = 12.23) was not significant, *t* (28) = 0.15, *p = 0*.*88*. A saccade was considered to be correct and entered into analysis if it landed in a square shaped AOI (Area of Interest) region measuring 2° (for perifoveal targets) or 4° (for peripheral targets) around the target.

**Fig 2:**
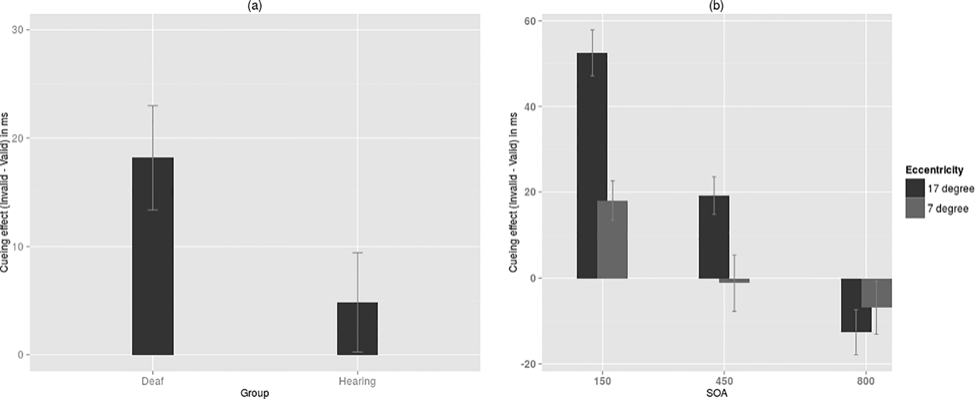
Oculomotor responses (a) Cueing effect for deaf and hearing groups (*p* = 0.05) (b) cueing effect at 150 ms, 450 ms and 800 ms SOA for both perifovea and periphery. The difference between perifovea and periphery is significant only at 150 ms and 450 ms SOA (*p <* 0.01). *Error bars represent ±1SD*.

Repeated measures ANOVA was performed on net cueing effect with group as a between-subjects factor and Eccentricity, SOA as within-subjects factors. Net cueing effect was calculated by taking the difference in saccade latency between invalid and valid trials (see S1 Table for mean values for saccade latency). We observed a significantly higher cueing effect in the deaf (M = 18.19 ms, SE = 4.69) compared to the normal hearing participants(M = 4.84 ms, SE = 4.69), *F*(1, 28) = 4.04, *p* = 0.054, *η* ^*2*^ = 0.13 (See Fig 2A). SOA had a strong effect on cueing effects, *F* (2, 56) = 65.84, *p* < 0.001, *η* ^*2*^ = *0*.*7*. There was facilitation at short and intermediate SOAs (M = 35.27 ms, SE = 3.49 and M = 9.03 ms, SE = 4.41 respectively), whereas inhibition at long SOA (M = -9.76 ms, SE = 4.12). There was a significant effect of Eccentricity, *F* (1, 28) = 11.32, *p* = 0.002, *η* ^*2*^ = *0*.*29*. *C*ueing effects were found to be larger at the periphery (M = 19.7, SE = 3.12) compared to the perifovea (M = 3.33, SE = 4.91). Eccentricity was found to interact significantly with SOA, *F* (2, 56) = 11.57, *p* < 0.001, *η* ^*2*^ = *0*.*29*. Pairwise compairsons using the LSD post-hoc tests showed that cueing effect at short and intermediate SOA was significantly larger (*p* = 0.001 and *p* = 0.006, respectively) at the periphery (M = 52.5 ms, SE = 5.07 and M = 19.23 ms, SE = 4.36 respectively) compared to the perifovea (M = 18.05 ms, SE = 4.64 and M = -1.17 ms, SE = 6.63 respectively) (Fig 2B). The difference in cueing effect between periphery (M = -12.63, SE = 4.67) and perifovea (M = -6.89, SE = 6.07) did not reach significance (*p* = 0.42) for 800 ms SOA. Group did not interact significantly with eitherEccentricity, *F* (1, 28) = 1.01, *p* = 0.30, *η* ^*2*^ = 0.04 or SOA, *F (*2, 56) = 2.31, *p* = 0.11, *η* ^*2*^ = 0.08. Further, the three way interaction between Group, Eccentricity and SOA also turned out to be non-significant, *F (*2, 56) = 1.64, *p* = 0.20, *η* ^*2*^ = 0.05.

### Error Saccades

Saccades that originated from a square of side measuring 2° around the fixation cross but did not land within the target area were considered as errors and entered into a mixed repeated measures ANOVA with group as a between subjects factor and eccentricity, Vvalidity and SOA as within-subjects factors. Group didn’t yield a significant effect, *F* (1, 28) = 0.19, *p* = 0.66, *η* ^*2*^ = 0.007 indicating that the deaf and normal participants made comparable errors in executing a saccade. The main effect of eccentricity was significant, *F* (1, 28) = 5.39, *p = 0*.*028*1, *η* ^*2*^ = 0.16. Participants made 3.3% more errors while making a saccade to the target at periphery (M = 9.4%, SE = 1.4) than when the target was at perifovea (M = 6.1%, SE = 1.2). Validity effect was also significant, *F* (1, 28) = 4.78, *p* = 0.037, *η* ^*2*^ = 0.15, with the error rate being higher for invalid trials (M = 8.6, SE = 1.3) compared to valid trials (M = 6.8, SE = 1.0). The effect of SOA was significant, *F* (2, 56) = 3.2, *p* = 0.048, *η* ^*2*^ = 0.37. Participants made more errors at short SOA (150 ms) (M = 8.7, SE = 1.3 compared to intermediate SOA (M = 7.4, SE = 1.3) and long (800 ms) SOA (M = 7.1, SE = 0.9). Eccentricity × SOA interaction was found to be significant, *F* (2, 56) = 5.99, *p* = 0.004 *η* ^*2*^ = 0.18. Pairwise comparisons showed that, at each SOA, percentage of errors was significantly higher (0.01 < p < 0.02) at periphery compared to perifovea.

### Block-wise analysis

Since there were a very large number of trials, to examine whether practice effects had an influence on the saccade latency, we decided to repeat the analysis taking blocks of trials as a factor. The experiment was divided into two halves and the two blocks were entered as factors in a mixed repeated measures ANOVA along with Eccentricity (7°, 17°), Validity (Invalid, valid) and SOA (150,450,800) as within-subject factors. Group (deaf, hearing) was the between-subjects factor. The main effect of block was significant, *F* (1, 28) = 7.35, *p* = 0.011, *η* ^*2*^ = 0.21. Participants were significantly faster in the second block (M = 297.21, SD = 9.13) compared to the first (M = 316.63, SD = 12.88). This suggests that there might have been some practice effects on saccade latencies. To examine whether the magnitude of IOR significantly differed in two blocks, we looked at the Block × Validity × SOA interaction which turned out not to be significant, *F* (2, 56) = 1.54, *p* = 0.22, *η* ^*2*^ = 0.05. Thus, block-wise analysis did not reveal any new results in terms of facilitation or inhibition for the saccade latency data.

### Response time

The manual response times were calculated independently of saccadic latencies.12.9% of trials for the deaf participants and 29.2% of trials for the normal hearing were excluded as the response times were below 100 ms or above 1200 ms. An independent samples t-test revealed that the percentage of excluded trials for the deaf group was significantly lower compared to the hearing group, *t* (28) = -2.98, *p* = 0.006. A manual response in a catch trial was considered as an error. The deaf and normal hearing participants made 2.29% and 0.14% catch trial errors respectively. However, this difference was not significant, *t* (28) = 1.0, *p* = 0.324.

**Fig 3:**
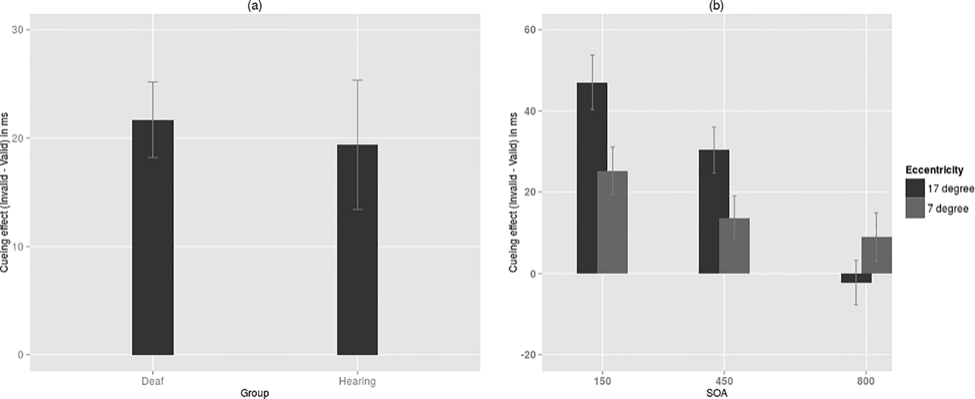
Manual responses. (a) Cueing effect for deaf and hearing groups (ns) (b) cueing effect at 150 ms, 450 ms and 800 ms SOA for both perifovea and periphery. The difference between perifovea and periphery is significant only at 150 ms and 450 ms SOA (*p <* 0.03). *Note*: *Error bars represent ±1SD*, *ns–not significant*.

Repeated measures ANOVA with group as the between-subjects factor and Eccentricity, SOA as within subjects factor was performed on the net cueing effect (See S2 Table for mean RTs). There was no main effect of group, *F* (1, 28) = 0.11, *p* = 0.74, *η* ^*2*^ = 0.004 (See Fig 3A). The main effect of eccentricity was significant, *F* (1, 28) = 5.11, *p* = 0.03, *η* ^*2*^ = 0.15. Cueing effect was higher when targets appeared at 17 degree (M = 25.06 ms, SE = 4.09) than at 7 degree (M = 16.01ms, SE = 3.9). SOA had a significant effect too, *F*(2, 56) = 16.12, *p* < 0.001, *η* ^*2*^ = 0.36. Facilitation was found at short, intermediate and long SOA (M = 36.16 ms, SE = 5.38; M = 22.02 ms, SE = 4.33 and M = 3.42 ms, SE = 4.64 respectively). There was a significant interaction between eccentricity and SOA, *F*(2, 56) = 7.43, *p* = 0.001, *η* ^*2*^ = 0.21 (Fig 3B). The cueing effect was significantly larger at periphery compared to perifovea only for 150 ms SOA (by 21.76 ms, *p* = 0.003) and 450 ms SOA (by 16.68 ms, *p* = 0.025). At 800 ms SOA, the difference in cueing effect between periphery and perifovea (11.27 ms) was not found to be statistically significant (*p* = 0.09). There were no significant interactions between Group and Eccentricity, *F (1*, *28)* = 0.84, *p* = 0.37, *η* ^*2*^ = 0.03 or Group and SOA, *F (2*, *56)* = 2.22, *p* = 0.12, *η* ^*2*^ = 0.07. Eccentricity × SOA × Group also didn't reach significance, *F* (2, 56) = 0.20, *p* = 0.82, *η* ^*2*^ = 0.01.

### Block wise analysis

A mixed repeated measures ANOVA was conducted on the response times with group as a between-subjects factor and Block (Block1,Block2), Eccentricity, **V**alidity and SOA as within-subjects factors. The main effect of Block was significant, *F* (1, 28) = 10.64, *p* < 0.001, *η* ^*2*^ = 0.27. Response times for the first half of the experiment were slower (M = 725.64, SE = 25.93) compared to the second-half (M = 656.72, SE = 25.63). Block did not interact significantly with any other factor.

## Discussion

In this study, we examined attention orienting in the deaf and the normal hearing individuals in a spatial cueing task with exogenous cues and measured oculomotor and manual responses for target detection. Both the deaf and the normal hearing participants were asked to make an eye movement and also give a manual response to targets that appeared at two spatial eccentricities following brief unpredictive spatial cues. We obtained two important results: Firstly, the cueing effect was larger for the deaf group compared to the normal hearing individuals. This effect was marginally significant, though. Secondly, we found significantly higher facilitation in target detections (at 150 ms and 450 ms SOA) at peripheral locations compared to perifovea regardless of the groups. There was facilitation for the short SOA and a minor inhibition for the long SOA for both deaf and hearing participants. "Facilitation" and "inhibition" refer to the difference in saccade latency between invalid and valid trials. This pattern of results is line with previous findings in spatial cueing studies where exogenous capture of attention has been shown to produce response benefit at short SOA but cost at long SOA. The deaf and normal hearing participants did not differ in overall saccadic latency. That is, the deaf did not programme faster saccades to the targets compared to the normal hearing. Besides, error analysis showed that deaf and normal hearing participants did not differ in the saccadic responses. However, we observed significantly higher errors for targets presented at the periphery as opposed to perifovea. Higher errors in the periphery can be explained due to lesser saccadic control to targets at the periphery that appear away from foveal vision. Further, there were higher errors on invalid trials compared to valid trials.

We obtained a group difference with the cueing effects for the ocular responses. Considering the fact that targets appeared at cued-at locations in valid trials, we assume that the deaf could orient attention to targets faster at cued locations. However, this difference was not modulated by the target eccentricity. Earlier, Parasnis and Samar [16], had observed that the deaf were faster in manual response latencies on invalid trials (to peripheral targets) suggesting that deaf may be better able to disengage and reorient attention from invalidly cued locations. Colmenero et al. [3], who had used a spatial cueing task had observed lower benefits (neutral RT–valid RT) and lower costs (Invalid RT–neutral RT) in the deaf compared to the normal-hearing in manual responses (Experiment 1). We would like to point out that these studies did not measure oculomotor responses like us. Hence, straight-forward comparisons are not possible. Considering that we used a dual-task, we did not observe group differences in net cueing on manual responses. Nevertheless, our results, at least with ocular responses, suggest an attentional orienting advantage for the deaf in the visual modality. Our results further indicate that perceptual detection of targets in space and visual spatial attentional mechanisms may be different processes in the deaf [26].

We also observed a significantly higher cueing effect at the periphery than perifovea irrespective of the groups, which is in line with previous studies. This effect was seen in both saccadic and manual responses. Bao and colleagues ([27], [28],[29],[14]) have consistently shown the effect of target eccentricity on IOR in a series of studies. For instance, Bao et al., [14] presented targets at 7 and 21-degree eccentricity in an exogenous cueing task which required manual responses. They observed that the magnitude of inhibition was much higher for the peripheral targets. According to Bao and colleagues, there is a functional dissociation between the attentional networks involved in responses to targets in peripheral and parafoveal/perifoveal regions of the visual field. Our data provides additional evidence that the peripheral effect on attentional orienting is not limited to inhibition but also extends to response facilitation observed at shorter SOAs.

The deaf were not necessarily faster in saccade or manual latencies as has been observed in some studies [18], [3], and [4]. Loke and Song [4] had observed faster responses to targets at the periphery in the deaf compared to hearing students on a detection task. Colmenero et al. [3] found overall faster RTs for deaf participants irrespective of SOA or cue validity. However, in contrast to these results, Bottari et al. [24] observed that deaf were not significantly faster in generating saccades compared to hearing individuals. Although we didn't find a significant difference in response latencies between the two groups, our results show that as far as ocular responses are concerned, deaf show an attentional advantage in orienting to targets. Several previous studies have found a visual/attentional advantage in the deaf, especially at the periphery [2], [3], [14].

It is quite possible that different types of tasks elicit different types of visuomotor behavior in the deaf. In an eye-tracking study using a pro- and anti-saccade task, Bottari et al. [24] observed faster saccade latencies to pro-saccade trials compared to anti-saccade trials. The authors concluded that the higher facilitation found in deaf was a result of faster reorienting of visual attention. Bottari et al., [24] also observed that deaf made greater errors in the anti-saccade trials than the pro-saccade trials compared to the hearing individuals. In our study, error saccades only revealed an effect of validity but not of the group. Participants made lesser errors when the cue and target location matched. It is important to note that we used a cueing paradigm, and it is possible that different paradigms engage attentional mechanisms differently. Colmenero et al. [3] measured manual responses with targets presented at the periphery on a spatial cueing task. They found inhibition at relatively short SOA (350 ms) which decayed faster and turned into facilitation at long SOA (850 ms) only in deaf but not in normal hearing (Experiment 2). This result is attributed to the ability of the deaf to disengage attention faster from periphery when compared to hearing individuals. In contrast, we found facilitation (at 150, 450 ms) which turned into inhibition (at 800 ms) in both the groups. For peripheral targets, there was a trend of higher facilitation at short SOA and lower inhibition at long SOA in deaf compared to normal hearing individuals. But these effects did not reach statistical significance.

In a methodological twist to the conventional cueing studies, we had also asked the participants to give a manual response on each trial as soon as they detected the targets. Most studies that measure saccadic responses in a spatial cueing paradigm ask participants only to detect the targets using eye movements. Thus, It is not clear whether these manual responses would show the characteristic patterns of facilitation and inhibition similar to the ocular responses. We found no group differences in response times or cueing effect. We did observe the characteristic facilitation at short SOA in both the groups for manual responses. However, we found no inhibition in deaf at long SOA in the manual responses. The effect of target eccentricity on cueing effect was found to be similar to saccadic responses. Participants showed higher facilitation at shorter SOAs (150 ms and 450 ms) at the periphery compared to perifovea. The interpretation of our results should be viewed considering the fact that our task was essentially a dual task. Participants first gave a saccadic response and then a manual response. Previously, saccadic and manual responses have been elicited in different blocks on attention tasks [22]. We would like to acknowledge that because of this dual task scenario, both the saccadic latencies and the manual RTs could have influenced one other. However, studies have shown that there appears to be a dissociation between sensory and motoric components of attentional orienting [23]. The premotor theory of spatial attention [30] (see also [16] for an overview) makes distinct predictions for cueing effects seen in manual and saccadic responses. The time course of appearance of facilitation and inhibition in the saccadic and the manual systems may differ [22]. It has also been shown that saccadic and manual responses arise in different neural processing pathways giving rise to different time scales of responses seen in tasks [31].Ocular responses to targets in a cueing task, measure the attentional/perceptual aspect of attentional orienting whereas manual responses in which eye movements are discouraged indicates the motoric and decisional aspects [32]. Future studies should explore attentional issues pertaining to the deaf in task situations requiring cross-modal responses.

### Conclusion

We observed a larger cueing effect in deaf compared to normal hearing. It is possible that because of their superior ability in perceiving visual signal the deaf were aware of the stimulation provided by the brief cue more than the normal hearing individuals. Our results also support the idea that the deaf show superior readiness to respond to visual stimuli at the periphery [33]. We have also shown that target eccentricity modulates the magnitude of cueing effect, regardless of groups. Higher facilitation for peripheral targets indicates that attentional mechanisms vary with respect to target locations in the visual field. In the end, we acknowledge that differences in design and methodology as well as response modality can influence patterns of orientation and disengagement of attention. Future attention studies on deaf should explore such alternatives more fully.

## Supporting Information

S1 Table Table giving mean saccade latency for all conditions. (DOCX)

S2 Table Table giving mean response time for all conditions. (DOCX)

S1 Raw Data Subject means for saccade latency data. (XLSX)

S2 Raw Data Subjects means for percentage of error saccades. (XLSX)

S3 Raw Data Subject means for block-wise saccade latency. (XLSX)

S4 Raw Data Subject means for Manual response time data. (XLSX)
