# Supplementary material for: Effect of Exogenous Cues on Covert Spatial Orienting in Deaf and Normal Hearing Individuals
Source: PLoS One. 2015 Oct 30;10(10):e0141324. doi: 10.1371/journal.pone.0141324 (PMC4627766; doi:10.1371/journal.pone.0141324)
Supplement: S1 Table — (DOCX) [file pone.0141324.s001.DOCX]

**Effect of exogenous cues on covert spatial orienting in deaf and normal hearing individuals**

***Supplementary information***

**Seema Prasad ^a†^, Gouri Shanker Patil^b^ & Ramesh Mishra^a^**

^a^Center for Neural and Cognitive Sciences, University of Hyderabad, Gachibowli, Hyderabad 500046, India.

^b^Ali Yavar Jung National Institute for the Hearing Handicapped, Manovikas Nagar
Secunderabad, 500 009, India

**†Corresponding author**

Seema Gorur Prasad

Center for Neural and Cognitive Sciences, Science Complex

University of Hyderabad

Hyderabad, India 500046

Email address: gp.seema@gmail.com

Phone number: +919480385444

**Table**

Table 1 : Mean and standard deviation of Saccade Latency.

| Eccentricity_Validity_SOA | Deaf  Mean (SD) | Hearing  Mean (SD) |
| --- | --- | --- |
| 7_N_150 | 291.88 (48.61) | 308.38 (71.56) |
| 7_N_450 | 275.88 (42.69) | 284.48 (58.26) |
| 7_N_800 | 288.2 (39.59) | 281.15 (59.97) |
| 7_Y_150 | 275.94 (41.05) | 288.21 (65.89) |
| 7_Y_450 | 271.98 (41.05) | 290.73 (58.48) |
| 7_Y_800 | 285.68 (28.64) | 297.44 (53.53) |
| 17_N_150 | 344.57 (47.57) | 353.16 (69.9) |
| 17_N_450 | 313.76 (39.97) | 324.84 (66.98) |
| 17_N_800 | 321.84 (42.30) | 320.50 (58.38) |
| 17_Y_150 | 281.04 (60.49) | 311.69 (60.97) |
| 17_Y_450 | 291.60 (30.44) | 308.54 (58.04) |
| 17_Y_800 | 320.76 (27.25) | 346.85 (52.79) |

***Note :*** *Eccentricity (7, 17 degree) ; Validity (No, Yes) ; SOA (150, 450, 800 ms) ; Group (Deaf, Hearing).*
