# Supplementary material for: Effect of Exogenous Cues on Covert Spatial Orienting in Deaf and Normal Hearing Individuals
Source: PLoS One. 2015 Oct 30;10(10):e0141324. doi: 10.1371/journal.pone.0141324 (PMC4627766; doi:10.1371/journal.pone.0141324)
Supplement: S2 Table — (DOCX) [file pone.0141324.s002.docx]

**Effect of exogenous cues on covert spatial orienting in deaf and normal hearing individuals**

***Supplementary information***

**Seema Prasad ^a†^, Gouri Shanker Patil^b^ & Ramesh Mishra^a^**

^a^Center for Neural and Cognitive Sciences, University of Hyderabad, Gachibowli, Hyderabad 500046, India.

^b^Ali Yavar Jung National Institute for the Hearing Handicapped, Manovikas Nagar
Secunderabad, 500 009, India

**†Corresponding author**

Seema Gorur Prasad

Center for Neural and Cognitive Sciences, Science Complex

University of Hyderabad

Hyderabad, India 500046

Email address: gp.seema@gmail.com

Phone number: +919480385444

**Table**

Table 1: Mean and standard deviation of RT for the manual responses

| Eccentricity_Validity_SOA | Deaf  Mean (SD) | Hearing  Mean (SD) |
| --- | --- | --- |
| 7_N_150 | 669.91 (99.69) | 712.53 (140.5) |
| 7_N_450 | 640.22 (106.13) | 694.71 (149.01) |
| 7_N_800 | 662.59 (109.77) | 705.24 (154.72) |
| 7_Y_150 | 652.69 (94.57) | 679.18 (137.83) |
| 7_Y_450 | 626.29 (99.92) | 681.28 (147.94) |
| 7_Y_800 | 647.8 (97.26) | 701.91 (137.74) |
| 17_N_150 | 712.47 (110.82) | 753.71 (136.09) |
| 17_N_450 | 682.40 (117.84) | 731.81 (138.11) |
| 17_N_800 | 692.98 (112.82) | 722.68 (142.34) |
| 17_Y_150 | 666.77 (103.25) | 705.32 (150.46) |
| 17_Y_450 | 650.94 (107.51) | 702.56 (135.24) |
| 17_Y_800 | 685.99 (95.69) | 734.09 (129.41) |

***Note :*** *Eccentricity (7, 17 degree) ; Validity (No, Yes) ; SOA (150, 450, 800 ms) ; Group (Deaf, Hearing).*
